# Improving the community-temperature index as a climate change indicator

**DOI:** 10.1371/journal.pone.0184275

**Published:** 2017-09-12

**Authors:** Diana Bowler, Katrin Böhning-Gaese

**Affiliations:** 1 Senckenberg Biodiversity and Climate Research Centre, Frankfurt am Main, Germany; 2 Institute of Ecology, Evolution & Diversity, Goethe University Frankfurt, Frankfurt am Main, Germany; Bangor University, UNITED KINGDOM

## Abstract

Climate change indicators are tools to assess, visualize and communicate the impacts of climate change on species and communities. Indicators that can be applied to different taxa are particularly useful because they allow comparative analysis to identify which kinds of species are being more affected. A general prediction, supported by empirical data, is that the abundance of warm-adapted species should increase over time, relative to the cool-adapted ones within communities, under increasing ambient temperatures. The community temperature index (CTI) is a community weighted mean of species’ temperature preferences and has been used as an indicator to summarize this temporal shift. The CTI has the advantages of being a simple and generalizable indicator; however, a core problem is that temporal trends in the CTI may not only reflect changes in temperature. This is because species’ temperature preferences often covary with other species attributes, and these other attributes may affect species response to other environmental drivers. Here, we propose a novel model-based approach that separates the effects of temperature preference from the effects of other species attributes on species’ abundances and subsequently on the CTI. Using long-term population data of breeding birds in Denmark and demersal marine fish in the southeastern North Sea, we find differences in CTI trends with the original approach and our model-based approach, which may affect interpretation of climate change impacts. We suggest that our method can be used to test the robustness of CTI trends to the possible effects of other drivers of change, apart from climate change.

## Introduction

Monitoring species response to climate change is an important challenge for ecologists and conservation managers. Generalized approaches that can be applied to population census data regardless of taxonomic groups are particularly useful as climate change indicators [1]. Such indicators allow for standardized comparisons of how different taxonomic groups and communities in different locations are responding. As ambient temperatures increase, the relative performance of warm-adapted species is predicted to exceed the relative performance of cool-adapted ones [2, 3]. This community change can be summarized by the community temperature index (CTI) [3], which is a community weighted mean—a commonly applied community summary variable [4]–of species’ temperature preferences. This change in community composition can be used as a generalized indicator of the realized impacts of temperature change on communities [5].

“Warming” or “thermophilization” of biological communities has been already recognized as one of the main fingerprints of climate change [1, 6]. This indicator is potentially more sensitive than other climate change indicators because it is based on changes in species’ population abundances [5, 7] and may be detectable anywhere within species’ ranges and not only at range limits. Positive trends in the CTI over past decades have been observed in bird and butterfly communities in Europe [5, 8, 9], birds in North America [10], and plants in Europe [11] and the tropics [12], although not consistently across all populations that have been examined [13]. Additionally, variation in the strength of CTI temporal trends among habitats have suggested interactions between climate change and land use change [6, 14, 15].

The CTI has similar advantages to related trait-based approaches that use the differential responses of species according to their traits to detect the impact of an environment driver. For instance, declines of species known to be sensitive to nutrient-rich conditions have indicated increased pollution. Such approaches have the advantage of naturally integrating the components of environmental change, at the relevant spatial and temporal scales, that are most important to the organism. Additionally, as a simple indicator to calculate and explain, it has a value in the communication of climate change research—representing a simple “bio-thermometer”–making it especially popular among bird and butterfly conservation organizations [16].

Despite these advantages, it has been recognized that interpretation of the CTI is not straightforward [17, 18]. Species’ temperature preferences often covary with other species attributes, particularly habitat preference [19]. Because of such covariation, it can be difficult to interpret the ecological significance of trends in the CTI, and specifically whether they are really caused by climate change or rather another driver of environmental change. For instance, forest bird species typically have cooler temperature preferences than farmland and urban species [17, 19]. Changes in land use can lead to changes in the abundances of species with different habitat preferences [20], which means that temporal trends in the CTI could be potentially due to land use change as well as climate change [17]. If such covariation is overlooked, CTI analysis could lead to misleading inferences on the rate at which species are responding to or being affected by climate change.

In a recent paper, Tayleur et al. [21] analyzed changes in the CTI of bird communities across Sweden and controlled for habitat type using a land cover map, but they did not control for any effects of *change* in habitat type, probably due to a general lack of long-term annual land cover data. Similarly, other proposed climate change indicators, such as the climate impact indicator [22], also do not consider potential effects of other drivers. We propose a novel model-based approach that uses regression to separate the effects of temperature preference from the effects of other species attributes, such as habitat preference, on population abundances, before the CTI is calculated. First, we use a simulation model to highlight the problem of the original CTI approach and to show how our proposed approach overcomes this. We then apply our approach to long-term population datasets of breeding birds in Denmark and demersal marine fish in the North Sea. Previous analysis indicates that abundance trends of European birds and North Sea fish are affected by climate change [5, 23] but in both cases additional factors are likely involved (agricultural intensification for birds; exploitation by fisheries for fish) and these factors may additionally affect the trends in the CTI. We compare the temporal trends in the CTI with the original and our new approach to examine the consequences for the interpretation of species response to climate change. Although we focus our approach on the CTI, we note that in fact it could be applied to other indices based on community weighted means, such as the community specialization index [24] or community weighted nutrient preferences [25].

## Materials and methods

The original community temperature index (CTI) is a community weighted mean of the temperature preferences of species in a community; in other words, the average temperature preference of the species in a community, in which each species’ temperature preference is weighted by its observed abundance [3]. The formula can be written as:

$$original\;CTI=\overset{n}{\underset{s=1}{\sum }}Temp.pref_{s}\times Relative.abundance_{s}$$

where the number of species in the community is *n* and each species (*s*) has a temperature preference and a relative abundance (the species’ abundance divided by the abundances of all species) in the community. Often, the analysis is additionally run using only presence/absence data for each species to examine CTI dynamics solely driven by species turnover rather than changes in abundance. In this case, the CTI is not weighted by relative abundance but rather 1 or 0 according to whether the species is present or absent, respectively. We focus this paper on the CTI that is weighted by species abundances but the problem and our approach equally applies to either CTI calculation.

### Model-based approach to CTI

We propose a model-based approach that calculates the CTI based on the proportion of population change between years that can be attributed to the temperature preferences of the species, and eliminates the annual change that can be linked to other species attributes (e.g., habitat breadth or preference) affecting species response to other drivers of change (e.g. land use change). The power of multiple regression is exploited to separate the annual effects of temperature preference from those of other species attributes. Multiple regression models of the relationships between species attributes and their long-term population trends have already been used to infer the impacts of climate change and other drivers [20]–here we extend this approach by using multiple regression models to estimate the annual effects of species’ temperature preferences on abundances to calculate a community-level summary variable, i.e., the CTI.

**Fig 1:**
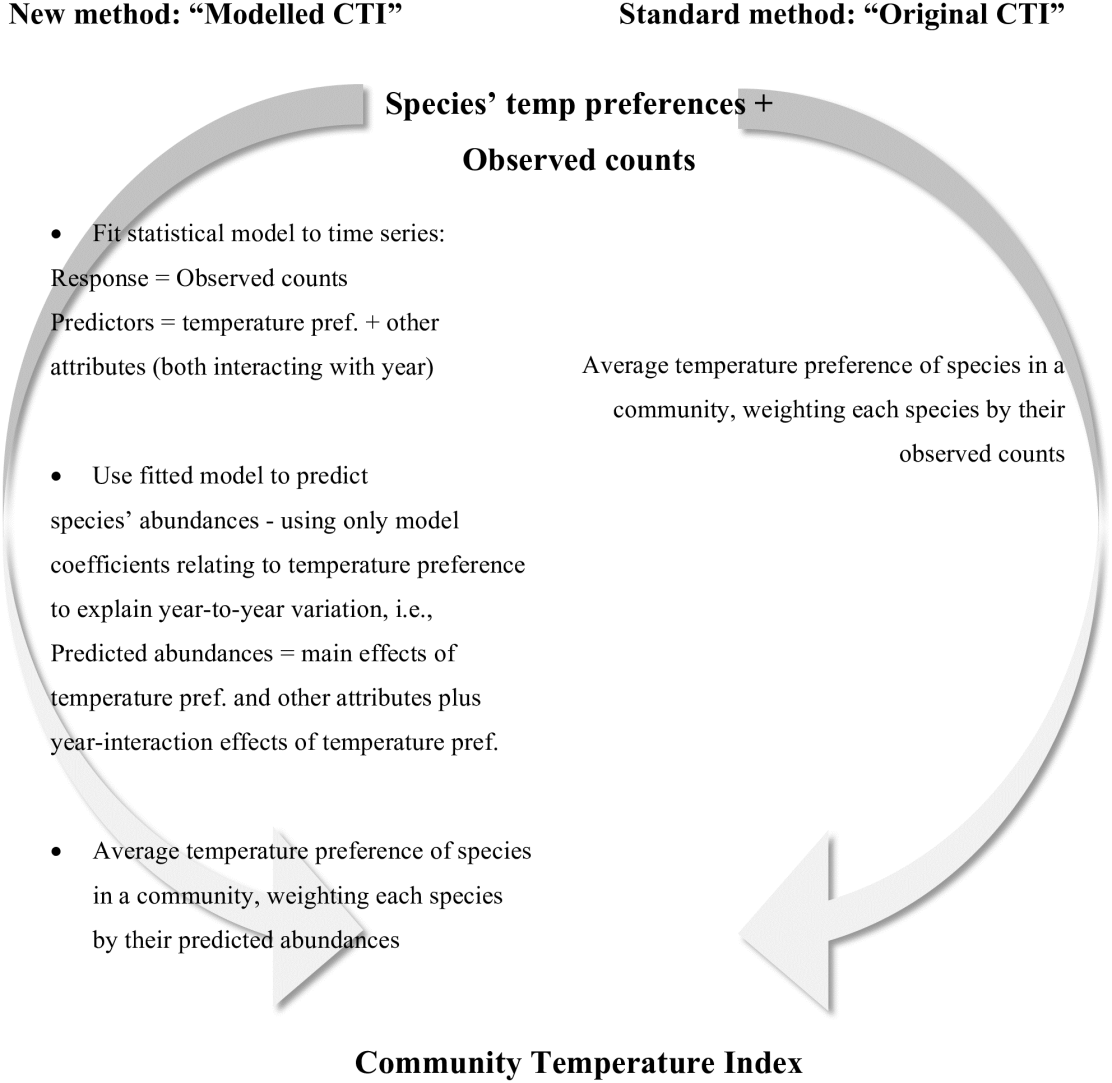
Outline of methods. The original method and our proposed new modelling method for calculating the CTI.

Specifically, the model-based approach to CTI calculation involves three main steps (Fig 1). First, we fit a statistical model to the time series of species’ observed counts using temperature preference and other species attributes (listed in each section below) as predictors. The effects of these predictors are allowed to interact with year (as a factor) so that their effects on species’ abundances can freely vary from one year to the next. Temporal autocorrelation can be accounted for by allowing for autocorrelated errors of consecutive time points [26]. As the strength of temporal autocorrelation is not assumed to vary over years, it does not interfere with the estimation of the fixed effects of the model that vary among years. Second, the statistical model is used to predict species’ abundances in each year. To remove the effect of other species attributes from year-to-year changes in abundances, this prediction step is only based on the main effects for all attributes and the year-interaction effects for temperature preference (i.e., the year-interaction effects for any other species attributes are not included). We also considered removing the main effects for other species attributes but left them in since we were only interested in the possible biases of changes over time (and their removal had in any case little effect). Finally, the CTI is calculated in the same way as in the original version except that these predicted abundances are used as the weighting, rather than the observed counts; hereafter, this is called the “modelled CTI”.

$$modelled\;CTI=\overset{n}{\underset{s=1}{\sum }}Temp.pref_{s}\times Predicted.relative.abundance_{s}$$

We note that an alternative approach to producing predicted abundances is to average 100 model predictions of the dataset with randomly sampled values of the other species attributes. This essentially averages out the effect of the other species attributes so that they no longer covary with the temperature preference effects. This achieved equivalent results (S1 Fig).

### Simulation model

We assumed a hypothetical ecological community of 50 species and tracked annual population change over a 20-year period. The abundance changes of each species over the years were affected by two species attributes: their temperature preference and their habitat breadth. Species’ temperature preferences and habitat breadths were drawn from random normal distributions with the same mean and variance. Species with warmer temperature preferences were assumed to have more positive population growth as a result of climate change. Species with broader habitat breadths were assumed to have more positive population growth as a result of being more tolerant to land use change. We ran three sets of simulation models. In the first set, only temperature preference affected species population growth, and these communities were used to calculate the “true CTI”. We regard this as the true CTI because it the CTI that results when only temperature preference, and hence climate change, affects the dynamics (S1 Table defines our terminology). In the second set of simulations, we assumed both temperature preference and habitat breadth affected growth and there was no covariation between species’ temperature preferences and their habitat breadths. In the third set, we assumed that both temperature preference and habitat breadth affected growth and that they negatively covaried (r = -0.6), i.e., species that were habitat generalists were cool-adapted—a scenario discussed by Stefanescu *et al*. [18] when comparing warm-adapted Mediterranean specialist butterflies with more cold-adapted and larger-ranged European generalist species. In all models, temperature preference and habitat breadth (when included) had the same magnitude of effects on population growth. Specifically, the abundance of each species in each year was:

$$\text{log}N_{s,t}\;\sim \;\text{log}N_{s,t-1}+b\;Temp.pref_{s}+b\;Habitat.breadth_{s}$$

where *N* is the abundance of species *s* in year *t* and *b* is the effect of temperature preference/habitat breadth on *N*. For simplicity, we assumed that the effects of the attributes remained constant over time.

For each community, we calculated the CTI for each year using both the original and our model-based approach as described above, using temperature preference and habitat breadth as predictors in the model (Fig 1). To check the robustness of our results to different assumptions about error, we ran each set of simulation models (apart from for the true CTI) with four kinds of error: (1) Poisson error, representing demographic stochasticity; (2) (Poisson +) overdispersion error, representing an unknown random (normally distributed) source of variation of twice the magnitude of the effect as habitat breadth/temperature preference; (3) (Poisson +) measurement error of temperature preference and (4) (Poisson +) measurement error of both temperature preference and habitat breadth. In both cases, measurement error was included as an additive effect of a random normal variable with mean of zero (measured vs actual, r = 0.7). We also checked assumptions about model structure by (1) including an interaction between temperature preference and habitat breadth—when no such interaction exists and (2) running models with and without a randomly generated variable as an additional predictor. Each set of simulations was run 500 times. Full parameters of the simulation are provided in S1 File, as well as an example script applying the approach to a dataset.

### European community time-series

#### Datasets

We applied our approach to two real-world datasets. The first dataset was from the Danish breeding bird survey coordinated by the Dansk Ornitologisk Forening and made available through their website (http://www.dof.dk) [27]. We used national abundance indices for terrestrial species that had been reliably surveyed during 1981–2013. Species affected by hunting/culling were excluded. Temperature preference was calculated as the average spring (April to June) temperature over the breeding range of each species, following others [28], so that both migrants and residents could be included (distribution data [29]; temperature data: EOBS 1961–1990 projected onto a 25 km grid [30]). However, CTI calculation is not linked with any particular method of temperature preference calculation. Species’ habitat preferences were divided into farmland and forest using the European Bird Census Council habitat classification (http://www.ebcc.info/wpimages/other/SpeciesClassification2011.xls), and further into urban species with data from Larsen et al. [31] when relative urban habitat use was greater than two. Because our aim here was more to illustrate the effects of covariation rather than calculate the actual CTI for specific communities, we excluded species from the dataset if they did not fall into one of these habitat groups (farmland, forest, urban) since most covariation with temperature preference was expected among these habitat types. The analysis presented in the main text was based on 36 species (S2 Fig shows the analysis including species from other habitat groups).

The second dataset was from demersal fish trawling surveys in the southern North Sea—part of the North Sea International Bottom Trawl Survey coordinated by the International Council for the Exploration of the Sea [32], made available through International Council for the Exploration of the Sea DATRAS database (http://www.ices.dk/marine-data/data-portals/Pages/DATRAS.aspx). We used catch per unit effort collected during winter (Jan to March) during 1980–2013 in “area 6”. We focused on relatively well-observed demersal species seen in at least 25% of the census years, comprising 34 species. One species was removed (*Syngnathus acus*) because it alone caused a strong increase in the CTI in the last census year and therefore could give a misleading picture—but its exclusion does not affect the general results (S3 Fig shows the analysis including this species). Temperature preference was calculated as the average annual temperature over each species range [distribution data: Ocean Biogeographic Information System/Global Biodiversity Information Facility; temperature data: bottom temperature from Aquamaps, which is based on the 2001 World Ocean Atlas annual means between 1990 and 1999 [33], projected onto a 50 km grid. We also compiled species information on maximum body length [34] and whether the species were exploited using the fishbase database [35], which could relate to the fishing pressure each species had experienced.

We assessed whether there was any covariation between species’ temperature preferences and the other species attributes using a linear model. To support the inclusion of the attributes, we also tested whether all attributes significantly affected species’ long-term population trends by testing the interaction between year (as a continuous variable) and each attribute on logged species’ abundances, accounting for temporally autocorrelated (of order 1) errors.

#### CTI calculation

We standardized the annual counts of each species relative to 100 in census year 1, so that all species began the time series with the same abundance index value. This was to avoid species that were dominant at the start of the time series from driving patterns in the CTI throughout all years. We calculated the original CTI using observed species counts, as already described. For the modelled CTI, we fitted a statistical model to the community dataset of species’ logged abundance indices with predictors including species’ temperature preferences as well as other species attributes (birds: habitat preference; marine fish: exploitation status and maximum length). We allowed the effects of the attributes (*Temp*.*pref* and *Attribute*) to interact with year (as a factor, *fYear*), as follows:

$$\begin{array}{l} \text{log}N_{s,t}\;\sim \;a+b_{i}fYear_{t}+b_{i}Temp.pref_{s}+b_{i}Attribute_{s}++b_{i:t+i}fYear_{t}:Temp.pref_{s}\\ +b_{i:t+i}fYear_{t}:Attribute_{s}+\rho \text{log}N_{s,t-1}+Sp_{s}+cYear_{s} \end{array}$$

where *a* and *b*_*i*_ (the number *I* will depend on the number of years) are estimated by the model. Temporal autocorrelation was accounted for in the model by estimating the residual correlation (*ρ*) among consecutive time points. Species effects were included as random intercepts (*Sp*), and species’ relationships between abundance and year (as a continuous variable, i.e., long-term trend, *cYear*) as random slopes. These sources of error were constrained to sum to zero. Interactions between the attributes were considered but not supported when comparing models with and without the interaction on the basis of the Watanabe-Akaike information criterion.

The fixed effects coefficients, plus species random intercepts, from the fitted regression model were used to predict the abundances of each species in each year, excluding the year-interaction effects of other species attributes. This means that year-to-year differences in predicted species’ abundances were only affected by their temperature preferences. The modelled CTI was then calculated using these predicted abundances. For predicting these abundances, we decided to keep in the main effect of all attributes (i.e., temperature preference as well as the other attributes) so that the intercept of the model in year 1 matched the original CTI and enable better comparison of differences on the change over time.

To check if the model reasonably captured the observed dynamics of the species, we also calculated a “modelled-uncorrected CTI” using the species’ predicted abundances using all the fixed effects of the model (species attributes, year, and their interaction) plus the species random intercepts. If the model is sufficient, the modelled but uncorrected CTI should be similar to the original CTI.

Finally, temporal trends in the CTI across the years were assessed by applying a locally weighted smoother (loess) to the time series of CTI for each CTI type: original, modelled-uncorrected and modelled.

#### Exploring the underlying mechanism

Previous analysis of the community temperature index has usually failed to recognize that positive temporal trends in the CTI could be due to declines of cold-adapted species, increases of warm-adapted ones or some combination of the two. We explored the relative roles of these two processes in each dataset by analysis of the average abundances of species in the upper and lower temperature preference quartiles.

## Results

### Simulations

**Fig 2:**
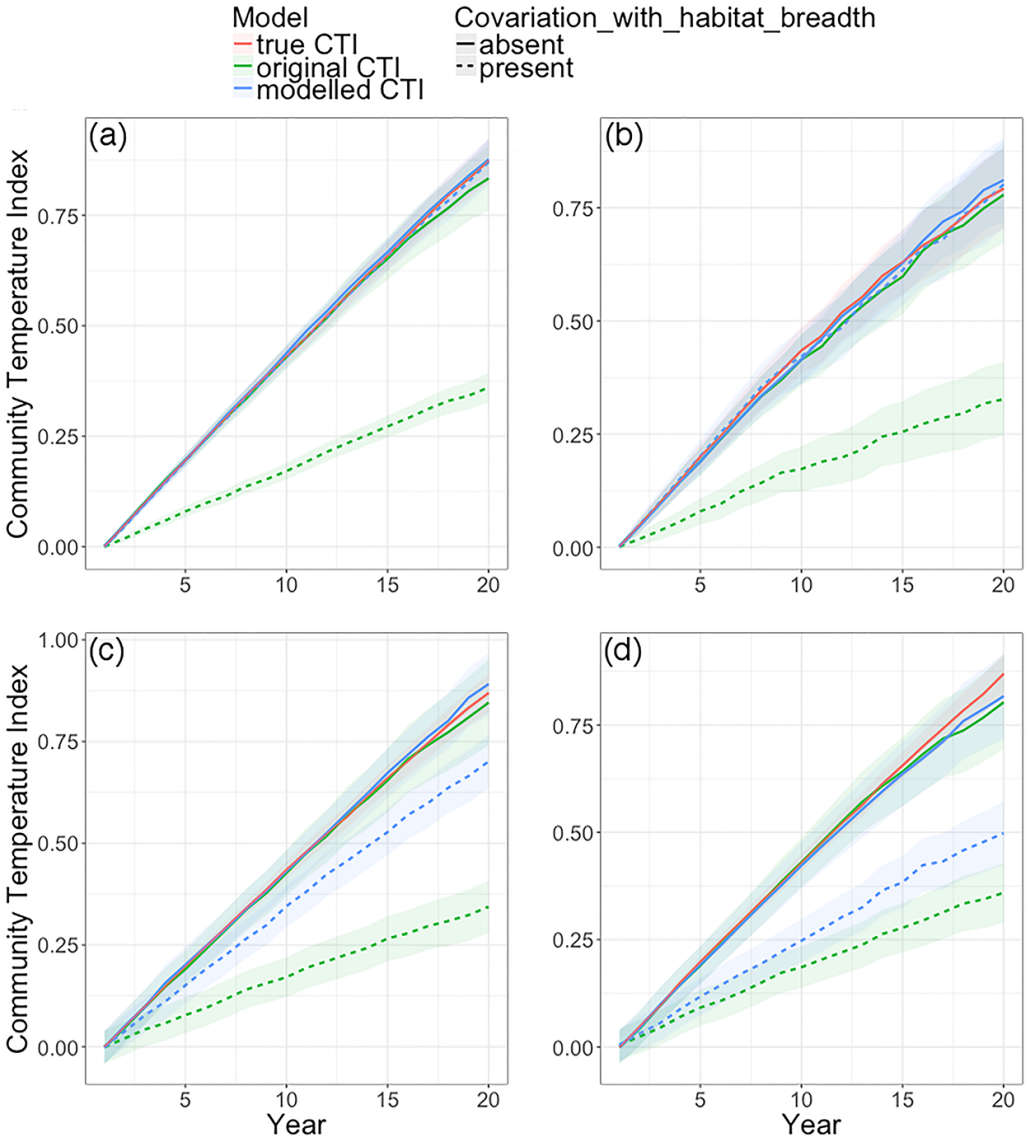
The effect of covariation between species’ temperature preferences and habitat breadths on the calculation of the community temperature index (CTI). The red line shows the “true CTI” from a community in which only temperature preference affected species population growth between years. For the remaining lines, habitat breadth was also assumed to affect population abundances. The dashed lines represent communities in which temperature preference and habitat breadth negatively covaried while the solid lines show the CTI from communities in which there was no such covariation. In these cases when habitat breadth also affected abundances, the CTI was either calculated as the “original CTI” (green lines) following current methodology or as the “modelled CTI” (blue lines) following our model-based approach. Error in the model arose due to Poisson error in (a); Poisson and overdispersion error in (b); Poisson and measurement error of temperature preference in (c), and Poisson and measurement error of temperature preference and habitat breadth in (d).

Our simulations aimed to test whether a model-based approach could overcome the problem of covariation among species attributes from biasing trends in the CTI, which increase with the strength of covariation (S4 Fig). When there was no covariation between temperature preference and habitat breadth, the original CTI performed as well as our modelled CTI in matching the “true” CTI (Fig 2a). In contrast, when there was negative covariation between temperature preference and habitat breadth, the original CTI performed poorly and, in this case, underpredicted the CTI. This was because the positive effect of temperature preference on population growth was obscured by warm-adapted species also having a narrow habitat breadth (i.e., high specialization) that had a negative effect of on population growth. However, the modelled CTI that excluded the effects of habitat breadth on species’ abundances was still able to match the true CTI (Fig 2a; blue, hatched line). The approach was found to be robust to overdispersion—because the extra variation was assumed to be not correlated with the species attributes (Fig 2b). When temperature preference was measured with some error, the modelled approach still performed better, i.e., its trend was closer to the true CTI, than using the original CTI method (Fig 2c), but with measurement error in both temperature preference and habitat breadth, the modelled CTI performed only slightly better than the original CTI when there was covariation (Fig 2d). The model was found to be similarly robust to model structure decisions, shown by the inclusion of superfluous model predictors (S5 Fig).

### Danish birds

**Fig 3:**
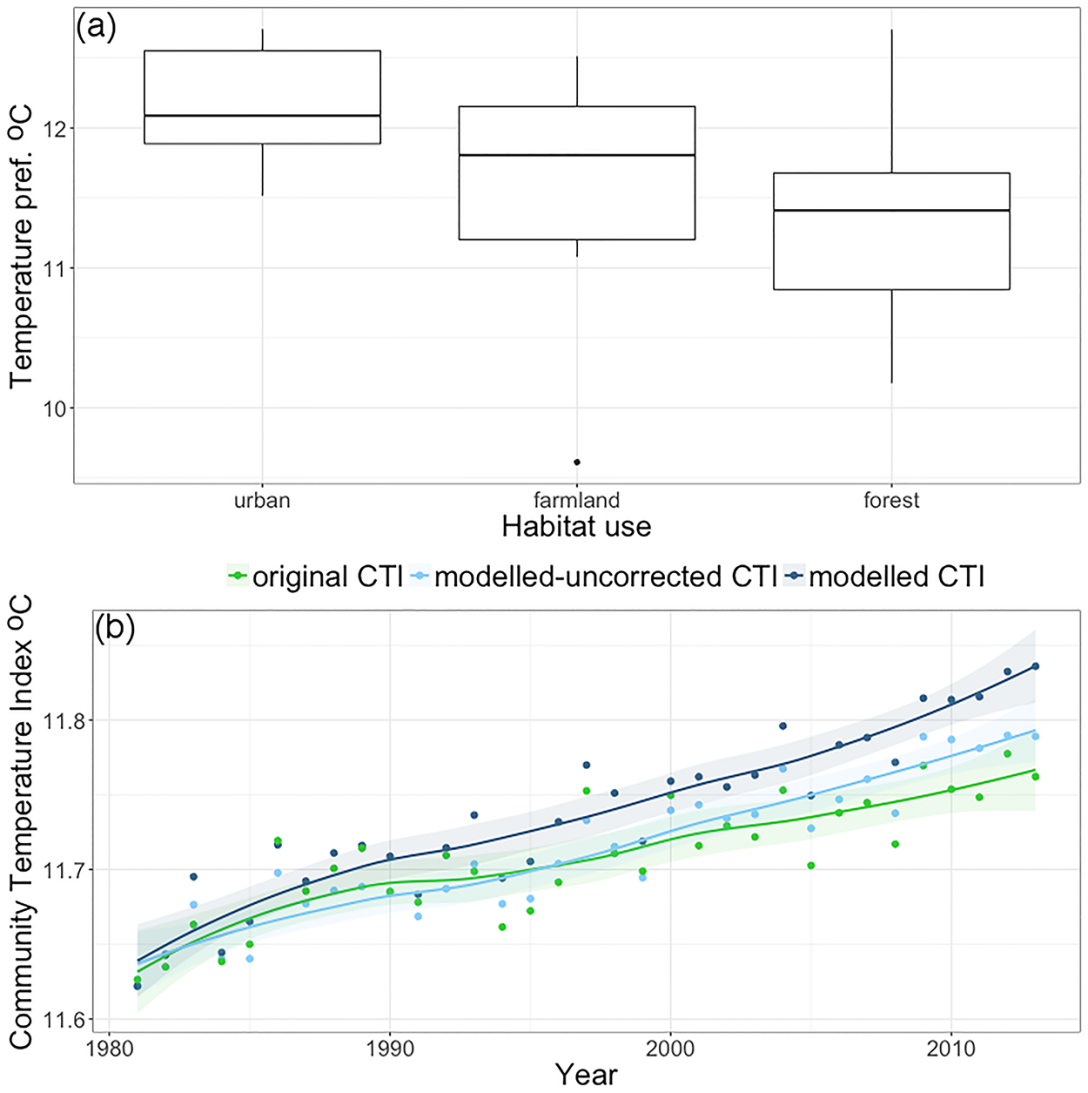
Temperature preference and CTI trends in the bird community. (a) Boxplots of temperature preferences for Danish breeding bird species (urban, farmland and forest species, n = 7, 13 and 16) according to their habitat preference. (b) Annual trends in the Community Temperature Index (lines are fitted loess ± 95% CI) of the same birds calculated as an average of species’ temperature preferences, with weighting in three ways: original CTI (weighted by observed counts); modelled–uncorrected CTI (weighted by model-predicted abundances); modelled CTI (weighted by model-predicted abundances that excluded the effects of habitat preference on abundances changes between years).

For the Danish birds, temperature and habitat preferences were not independent: urban birds tended to have warmer temperature preferences, followed by farmland birds, and finally forest species (Fig 3a, F_2,33_ = 4.18, P = 0.02). Both temperature preference and habitat preference explained species’ long-term trends: warm-adapted species have increased more than cool-adapted ones and forest birds have increased more than farmland birds (Table 1).

**Table 1:** Effects of species attributes on species’ long-term population abundance trends. Attributes were tested in a multiple regression model for each taxa.

Taxa	Species attribute	Estimate	SE	95% CI
Danish birds (n = 36)	Temperature preference (°C)	0.010	0.002	0.005, 0.015
	Habitat (Forest vs Farmland)^$^	0.015	0.004	0.007, 0.022
North Sea demersal fish (n = 32)	Temperature preference (°C)	0.0101	0.0021	0.006, 0.014
	Fishing interest (highly commercial vs no interest)^$^	-0.0309	0.0081	-0.047, -0.015

^$^we only present the pairwise comparison with the largest difference.

Consistent with these long-term trends, whatever the CTI calculation methodology, there was a clear signal of climate change on the community composition of Danish birds, demonstrated by the increase in the CTI over time (Fig 3b). The modelled—uncorrected CTI captured well the dynamics of the original CTI indicating that the model fitted the community dynamics. For the modelled CTI, which removed the confounding effects of habitat preference, the CTI temporal trend tended to be steeper than the original CTI (Fig 3b).

### North Sea demersal fish

**Fig 4:**
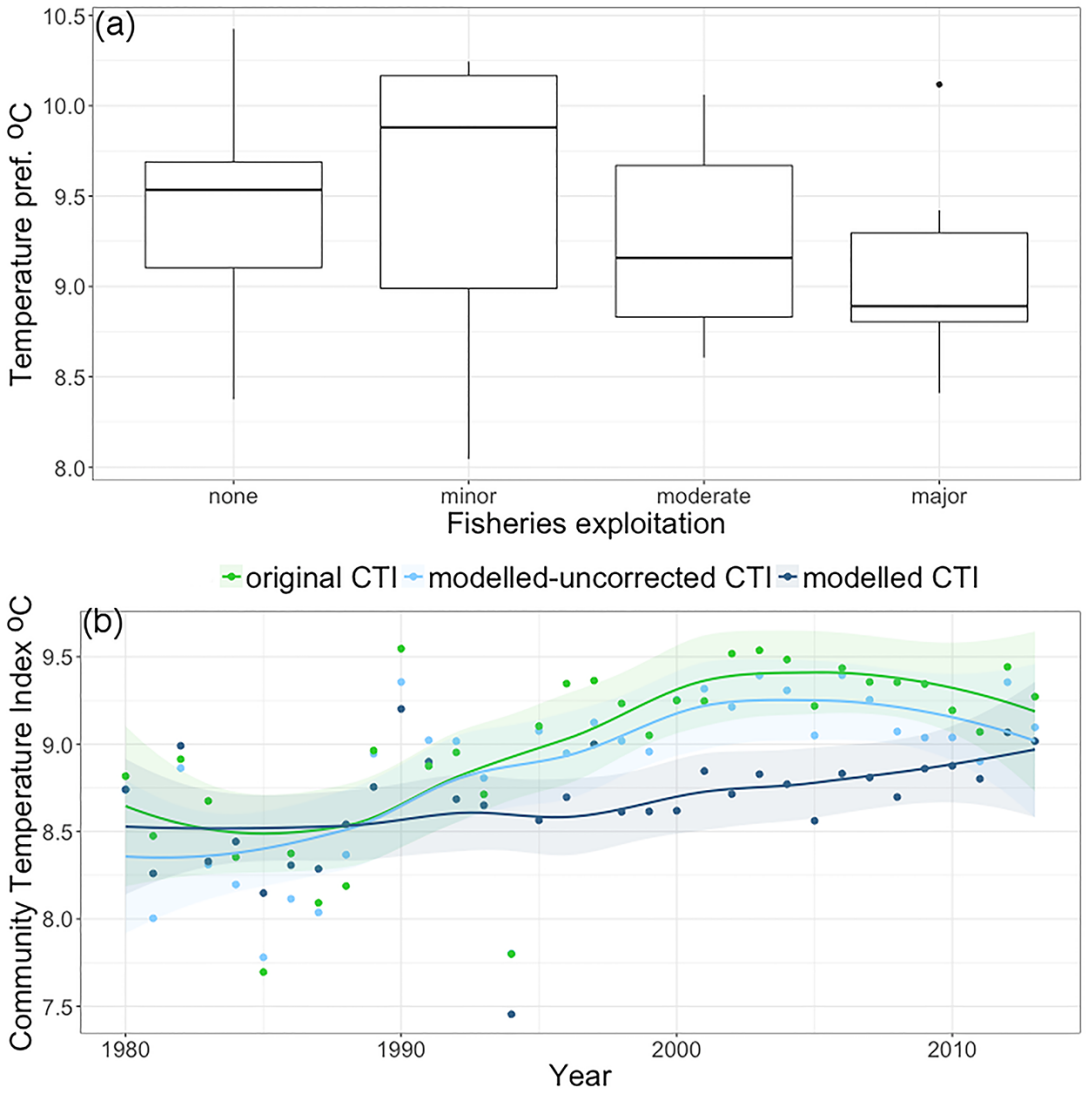
Temperature preference and CTI trends in the demersal fish community. (a) Boxplots of temperature preferences for demersal fish from the southeastern part of the North Sea according to their fisheries exploitation (left to right, n = 9, 7, 9 and 7). (b) Annual trends in the Community Temperature Index (lines are fitted loess ± 95% CI) of the same fish calculated as an average of species’ temperature preference, with weighing in three ways: original CTI (weighted by observed counts); modelled–uncorrected CTI (weighted by model predicted abundances); modelled CTI (weighted by model predicted abundances that excluded the effects of commercial interest and log maximum body length on changes in abundance between years).

Temperature preferences of North Sea demersal fish tended to vary with fishing exploitation. Although the pattern was not significant (F_3,29_ = 0.613, P = 0.6), fish that were moderately or highly commercially exploited tended to prefer cooler temperatures than fish of less interest to fisheries (Fig 4a). However, a general relationship can be hypothesized assuming that larger fish are of greater commercial interest than small fish, and that larger fish tend to be more northerly distributed (and hence have a cooler temperature niche) than more southerly distributed fish [36]. Fish under greater commercial exploitation showed lower population trends than fish of less commercial interest (Table 1).

The modelled—uncorrected CTI captured reasonably well the dynamics of the original CTI indicating that the model fitted the community dynamics (Fig 4b). All CTIs suggested that the community shifted to more warm-adapted species but they differed in the extent to which this occurred. The modelled CTI that excluded the effects of commercial interest and body size showed a weaker temporal trend (Fig 4b).

### Exploration of mechanism

**Fig 5:**
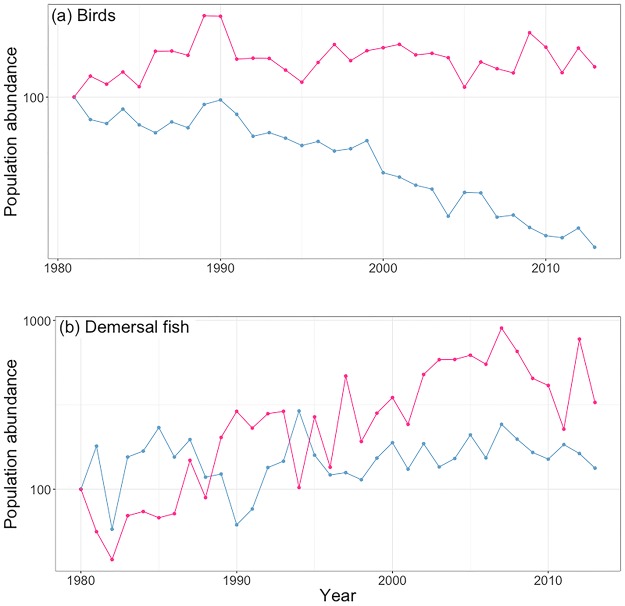
Population abundances of warm and cool-adapted species. Average (geometric mean) population abundances of warm (red lines) and cold-adapted (blue lines) species, i.e., species in the upper and lower quartiles of temperature preference, of (a) Danish birds and (b) demersal fish in the southeastern North Sea.

Analysis of Danish bird data using both the original data as well as the modelled data showed that the positive CTI trend was mostly due to declines of cold-adapted species (Fig 5a). In contrast, in the demersal fish community, the positive CTI trend was due to increases of warm-adapted species (Fig 5b).

## Discussion

Indicators of climate change impacts are only useful when they are unaffected by other drivers of biodiversity change. Previously, temporal trends in the CTI have been used to highlight the impacts of climate change on communities of different taxa [5]. Although other approaches can be applied to detect climate change impacts on populations (such as relationships between ambient temperatures and population abundance), the CTI provides a simple community-level indicator of changes in community composition. We show how trends in the community temperature index can be affected by covariation between temperature preferences and other species attributes, which may bias interpretation of the impacts of climate change. We have proposed and applied a new method that could verify the robustness of trends in the CTI and allow greater confidence that temporal trends in the CTI reflect the impact of climate change and not those of other drivers. In both real-world datasets that we analysed, species’ temperature preferences tended to covary with other attributes that probably affected response to drivers unrelated to climate change, and in turn this covariation influenced the strength of the temporal trend in the CTI.

In the demersal marine fish community, species with warm temperature preferences tended to be those of least interest from the fisheries industry, probably because they were usually smaller [30]. Thus, both increased temperatures and increased fishing pressure could lead to positive trends in the CTI of a community. Since both drivers likely operated for this community, the effect of covariation between temperature preference and exposure to fisheries was to bias the CTI trend upwards because warm-adapted species are favored by both drivers. Thus, the long-term increase in the original CTI was probably caused only partly by climate change, but potentially also by increases in fish species that were not commercially exploited, which may be an indirect effect of fishing [37]. Because even fish not the target of fisheries are often affected by fishing activities, e.g. by being caught as bycatch, the effect of commercial fisheries interest may not entirely capture the effect of fisheries on fish communities [38]. However, our results suggest greater uncertainty regardless of the processes underlying the increased CTI of demersal fish communities in the North Sea.

The effect of covariation in the bird dataset of farmland, forest and urban species was much weaker although the long-term trend in the CTI was slightly steeper after correction for habitat preference. In fact, the effect was even weaker when including the whole bird community for which there was available data (S2 Fig). Thus, the original CTI is not necessarily strongly biased when there is covariation among attributes. The major change in the bird community has been the decline of farmland birds, which can be linked to changes in agricultural practices [39]. This differs from the trends seen in other birds, especially forest species. However, farmland species tended to have warmer temperature preferences than forest species, and thus are expected to benefit more from climate change. Because climate change favors species that are negatively affected by land use change, the CTI trend was more positive after removing the effects of species’ habitat preferences.

Knowledge of the relationship between species temperature preference and other attributes, and how these attributes mediate species response to environmental drivers, can be used to predict the direction of potential bias in the CTI due to climate change-unrelated drivers. Critically, the direction of the bias depends on whether species favored by climate change are also favored or disfavored by the most significant other drivers. For example, as we found in our marine fish analysis, both climate change and fishing may benefit (or least less negatively affect) smaller, warm-adapted species. Since the same species are favored by both drivers, trends in the CTI could be biased upwards by alternative drivers (exploitation in the case), and thus the CTI trend would be less steep on correction. A recent study of Neotropical forest and farmland birds found that species likely to better cope with increased agricultural area were also those that tolerate a drier climate caused by climate change [40]. Thus, in this case, positive trends in the CTI could be driven by either climate change or habitat conversion. However, in European bird populations, farmland birds populations have warmer temperature preferences than other birds [17], but have been more strongly affected by agricultural intensification [20]. Thus, in this case, species favored by climate change are more negatively affected by significant other drivers, and the original CTI may be biased downwards.

Error in the estimation of species attributes is an important consideration in the calculation of the CTI. Devictor et al. [41] previously argued that trends in the CTI should be relatively robust to such error because the estimation of species’ temperature preferences only needs to reflect the gradient in preferences rather than determine their exact optimum temperature values. We found in our simulation analysis that the original and model-based CTI approach was somewhat sensitive to the accuracy of data available on species’ temperature preferences and other attributes, but only when there was covariation between species attributes. Unsurprisingly, the model-based approach was less able to correct for covariation when the underlying data was poor; but at least it did not perform worse than making no attempt to correct for it.

Like any approach that uses the effects of species traits/attributes as proxies of the impacts of environmental drivers, its success depends on how well the species traits/attributes are linked with the environmental drivers. There is theoretical and empirical support that temperature preference is an important determinant of species responses to climate change; however, some climate change impacts may be more dependent on other species attributes [42]. Similarly, the success of our CTI correction depends on how well species’ habitat preferences can be linked with the effects of land-use change. Certainly, the effect of farmland use by birds has successfully highlighted the impacts of agricultural intensification in European bird populations [20]: however, not all land-use changes might be as easily focused on species with a specific habitat preference.

We would recommend a number of steps when applying our approach. Our approach requires an understanding of what attributes covary with temperature preference as well as of the factors affecting population dynamics. Additionally, the number of species attributes that could be added to the model will be limited by the number of species with different attributes in the community. We first recommend investigation of which species attributes are significantly linked with species’ long-term population trends when tested together in a multiple regression model because it is these attributes that are likely to bias temporal patterns in the CTI. Secondly, the covariation among these attributes should be examined; the larger the covariation, the more likely the bias. However, our approach obviously cannot be used if there is very strong covariation (e.g., if |r|>0.7) between temperature preference and another predictor because including both in the statistical model would introduce collinearity [43]. We would also encourage users to examine the distribution of trait values across species in the community. The effect of covariation on CTI bias may depend on trait distribution; for instance, if there is high imbalance in the number of species in different habitat groups, any covariation between temperature preferences and different habitats preferences is less likely to be important.

A steep increase of the CTI over time is not suggestive that a taxonomic group will persist in the face of climate change. Positive trends could be a result of increases of warm-adapted species, perhaps by immigrating species or greater survival /reproductive success of species already members of the community. Equally possible, positive temporal trends of the CTI could result from declines or local extinctions of cold-adapted species. Despite helping to understand the mechanism and potential consequences of community change, these two possibilities are often over-looked in CTI analysis. We extended our analysis to examine the cause of the CTI trends in our datasets. We found that the positive temporal trends seen in the birds and fish were driven by very different mechanisms. Increases in the bird CTI were driven by decreases of cold-adapted species, suggesting that climate change could reduce the size of the bird community. In contrast, increases of warm-adapted species drove the CTI trend of the marine fish, which indicates that some species are benefiting from the warmer temperatures due to climate change.

The CTI is but one of several indices based on a community-weighted mean. For instance, the community specialization index (CSI) is a weighted average of the niche breadth of species in a community and has been used to indicate the extent to which communities have become more dominated by generalists [24, 44]. Such indices that are also based on species attributes might be similarly affected by covariation among species attributes, and our approach may also be considered in these cases.

Our findings indicate that inferences of climate change impacts based on the CTI to date might have been biased by the effect of other environmental drivers such as land use change and exploitation. Our proposed approach can check the robustness of trends in the CTI to other drivers. Although our model-based approach is slightly more complicated than the original approach, it uses relatively straightforward linear modelling. The CTI remains a simple tool to test and visualize whether climate change has affected a group of organisms, and to allow a comparison of the effects among different organisms or in different regions.

## Supporting information

S1 Fig The effect of using different methods to obtain the modelled CTI. In (a) the predicted abundances of each species, without the confounding effect of habitat breadth, was calculated in the same way as presented in the main text (i.e., dropping model coefficients of year X habitat breadth to predict abundances). In (b) an alternative but equivalent approach is presented that entails averaging over model predictions for randomly sampled values of the habitat breadth to produced predicted abundances, which are then used to calculate the modelled CTI. For further details see the legend of Fig 2 in the main text. Code to follow the approach for (b) is shown below.
(DOCX)

S2 Fig The effect of including bird species from all habitat types, not just farmland, urban and forest. The same analysis is presented as shown in Fig 3 but using more species. Here, 65 species were included and those not falling into one of the aforementioned habitat types were classed as having “other” habitat preference as their species attribute. We still excluded species that were not annually censused 1981 onwards and if they were affected by hunting or culling.
(DOCX)

S3 Fig The effect of including an outlier species (*Syngnathus acus*) on the results presented in Fig 4. The original CTI value in the last census year is shown to be strongly influenced by the abundance of this species. However, the general patterns remain the same: the modelled CTI is lower than the original CTI, especially since 1995.
(DOCX)

S4 Fig Analysis of the factors affecting the bias in the CTI. Bias (difference between the true CTI and the original CTI—see S1 Table for explanation of terms) when temperature niche correlates with habitat breadth, assuming the same dynamics as presented in the simulations of the main text. The results are the mean and 95% of the bias from 500 simulation runs. The CTI bias is found to increase with the correlation with habitat breadth; to increase but at a decelerating rate with the effect size of the attributes, and to be unaffected by the number of species in the community.
(DOCX)

S5 Fig The effectiveness of the modelled CTI approach is robust to different sorts of assumptions. (a) for comparison, the original simulation results shown in Fig 2a; (b) the effect of including a 3-way interaction between temperature niche, habitat breadth and year in the statistical model to obtain the predicted abundance when no such interaction existed in the underlying dynamics of the simulated species; (c) the effect of included an additional randomly generated variable in the statistical model to obtain the predicted abundance when it has no true effect on species’ abundances; (d) the effect of assuming time-varying effects of temperature niche and habitat breadth (effect sizes assumed to increase and decrease over time respectively) in the underlying dynamics of the simulated species and (e) the effect of including random variation in the density-dependence of the underlying dynamics of the simulated species.
(DOCX)

S1 Table Definition of CTI terminology used in the paper. (DOCX)

S1 File R script for the simulations and application to a real-world dataset. (DOCX)
