# Supplementary material for: Improving the community-temperature index as a climate change indicator
Source: PLoS One. 2017 Sep 12;12(9):e0184275. doi: 10.1371/journal.pone.0184275 (PMC5595310; doi:10.1371/journal.pone.0184275)
Supplement: S1 Fig — In (a) the predicted abundances of each species, without the confounding effect of habitat breadth, was calculated in the same way as presented in the main text (i.e., dropping model coefficients of year X habitat breadth to predict abundances). In (b) an alternative but equivalent approach is presented that entails averaging over model predictions for randomly sampled values of the habitat breadth to produced predicted abundances, which are then used to calculate the modelled CTI. For further details see the legend of Fig 2 in the main text. Code to follow the approach for (b) is shown below. (DOCX) [file pone.0184275.s001.docx]

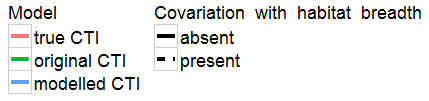


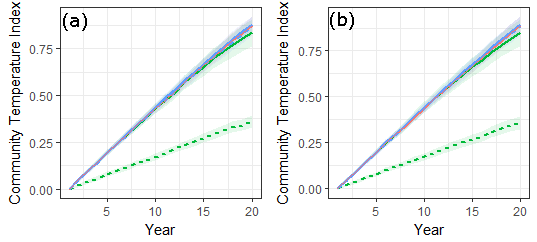


**S1 Fig.** **The effect of using different methods to obtain the modelled CTI.**

In (a) the predicted abundances of each species, without the confounding effect of habitat breadth, was calculated in the same way as presented in the main text (i.e., dropping model coefficients of year X habitat breadth to predict abundances). In (b) an alternative but equivalent approach is presented that entails averaging over model predictions for randomly sampled values of the habitat breadth to produced predicted abundances, which are then used to calculate the modelled CTI. For further details see the legend of Fig 2 in the main text. Code to follow the approach for (b) is shown below.

Alternative code to get modelled abundances randomizing the effect of potentially confounding species attributes. See the “Bowler_SOM_CTI_code.doc” for the whole script.

#########################################################################

#get model fits just using temperature niche as the coefficient affecting #year-to-year change

mypreds<-matrix(ncol=1000,nrow=1000)

for(i in 1:1000){

species.dfR<-species.df

species.dfR$HS<-sample(species.dfR$HS)

dfR<-df

dfR$HS<-species.dfR$HS[match(dfR$Species,species.dfR$Species)]

#modelR<-lm(Count~fYear*HS+fYear*TNunc,data=dfR)

modelR<-lm(Count~fYear*HS+fYear*TN,data=dfR)

outFE<-data.frame(model.matrix(modelR,dfR))

outFE<-as.matrix(outFE)

coefFE<-as.matrix(inla1$summary.fixed[,1],ncol=1)

mypreds[,i]<-outFE%*%coefFE

}

df$mypreds<-rowMeans(as.data.frame(mypreds))

##also get species intercepts term and add it on

temp<-inla1$summary.random[["Species.int"]][,c("ID","mean")]

df$speciesint<-temp$mean[match(df$Species.int,temp$ID)]

df$mypreds<-df$mypreds+df$speciesint

#get corrected CTI based on these corrected fits

#CTIanalysis2_corr<-#ddply(df,.(Year),summarise,CTI=wtd.mean(TNunc,weights=exp(mypreds)))

CTIanalysis3_corr<-ddply(df,.(Year),summarise,CTI=wtd.mean(TNunc,weights=exp(mypreds)))

####################################################################
