# Supplementary material for: Improving the community-temperature index as a climate change indicator
Source: PLoS One. 2017 Sep 12;12(9):e0184275. doi: 10.1371/journal.pone.0184275 (PMC5595310; doi:10.1371/journal.pone.0184275)
Supplement: S2 Fig — The same analysis is presented as shown in Fig 3 but using more species. Here, 65 species were included and those not falling into one of the aforementioned habitat types were classed as having “other” habitat preference as their species attribute. We still excluded species that were not annually censused 1981 onwards and if they were affected by hunting or culling. (DOCX) [file pone.0184275.s002.docx]

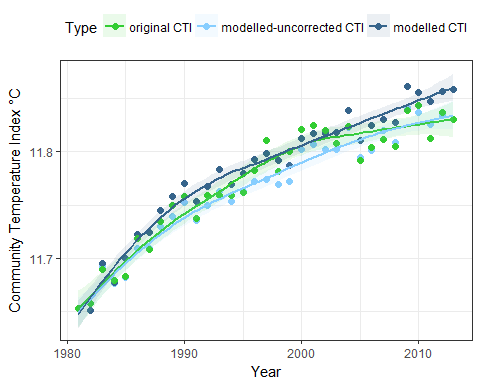


**S2 Fig**. **The effect of including bird species from all habitat types, not just farmland, urban and forest.**

The same analysis is presented as shown in Figure 3 but using more species. Here, 65 species were included and those not falling into one of the aforementioned habitat types were classed as having “other” habitat preference as their species attribute. We still excluded species that were not annually censused 1981 onwards and if they were affected by hunting or culling.
