# Supplementary material for: Improving the community-temperature index as a climate change indicator
Source: PLoS One. 2017 Sep 12;12(9):e0184275. doi: 10.1371/journal.pone.0184275 (PMC5595310; doi:10.1371/journal.pone.0184275)
Supplement: S3 Fig — The original CTI value in the last census year is shown to be strongly influenced by the abundance of this species. However, the general patterns remain the same: the modelled CTI is lower than the original CTI, especially since 1995. (DOCX) [file pone.0184275.s003.docx]

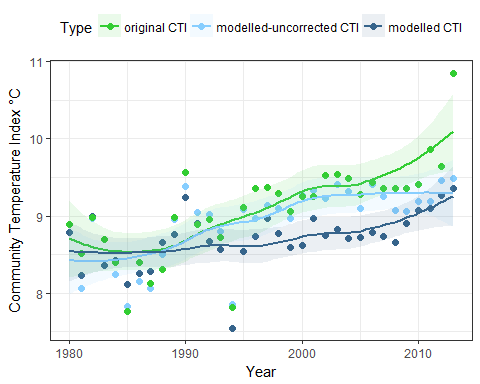


**S3 Fig**. **The effect of including an outlier species (*Syngnathus acus*) on the results presented in Fig 4.**

The original CTI value in the last census year is shown to be strongly influenced by the abundance of this species. However, the general patterns remain the same: the modelled CTI is lower than the original CTI, especially since 1995.
