# Supplementary material for: Improving the community-temperature index as a climate change indicator
Source: PLoS One. 2017 Sep 12;12(9):e0184275. doi: 10.1371/journal.pone.0184275 (PMC5595310; doi:10.1371/journal.pone.0184275)
Supplement: S4 Fig — Bias (difference between the true CTI and the original CTI—see S1 Table for explanation of terms) when temperature niche correlates with habitat breadth, assuming the same dynamics as presented in the simulations of the main text. The results are the mean and 95% of the bias from 500 simulation runs. The CTI bias is found to increase with the correlation with habitat breadth; to increase but at a decelerating rate with the effect size of the attributes, and to be unaffected by the number of species in the community. (DOCX) [file pone.0184275.s004.docx]

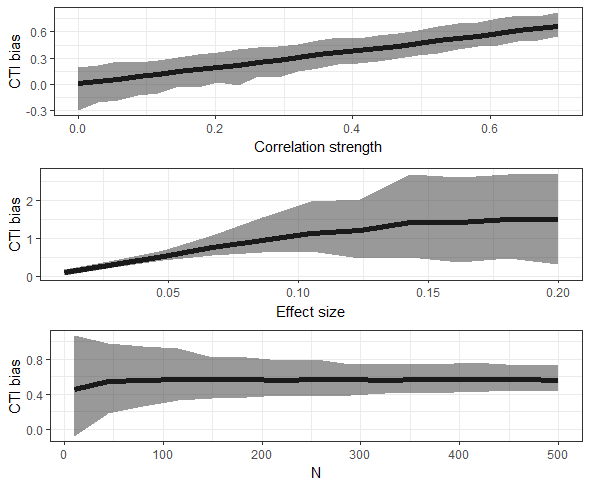


**S4 Fig**. **Analysis of the factors affecting the bias in the CTI.**

Bias (difference between the true CTI and the original CTI – see Table S1 for explanation of terms) when temperature niche correlates with habitat breadth, assuming the same dynamics as presented in the simulations of the main text. The results are the mean and 95% of the bias from 500 simulation runs. The CTI bias is found to increase with the correlation with habitat breadth; to increase but at a decelerating rate with the effect size of the attributes, and to be unaffected by the number of species in the community.
