# Supplementary material for: Improving the community-temperature index as a climate change indicator
Source: PLoS One. 2017 Sep 12;12(9):e0184275. doi: 10.1371/journal.pone.0184275 (PMC5595310; doi:10.1371/journal.pone.0184275)
Supplement: S5 Fig — (a) for comparison, the original simulation results shown in Fig 2a; (b) the effect of including a 3-way interaction between temperature niche, habitat breadth and year in the statistical model to obtain the predicted abundance when no such interaction existed in the underlying dynamics of the simulated species; (c) the effect of included an additional randomly generated variable in the statistical model to obtain the predicted abundance when it has no true effect on species’ abundances; (d) the effect of assuming time-varying effects of temperature niche and habitat breadth (effect sizes assumed to increase and decrease over time respectively) in the underlying dynamics of the simulated species and (e) the effect of including random variation in the density-dependence of the underlying dynamics of the simulated species. (DOCX) [file pone.0184275.s005.docx]

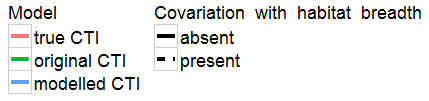


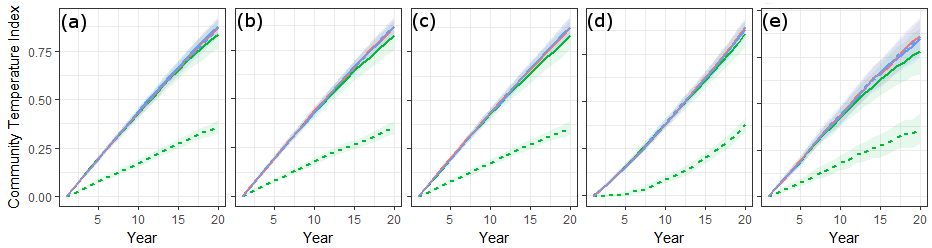


**S5 Fig**. **The effectiveness of the modelled CTI approach is robust to different sorts of assumptions.**

(a) for comparison, the original simulation results shown in Fig. 2a; (b) the effect of including a 3-way interaction between temperature niche, habitat breadth and year in the statistical model to obtain the predicted abundance when no such interaction existed in the underlying dynamics of the simulated species; (c) the effect of included an additional randomly generated variable in the statistical model to obtain the predicted abundance when it has no true effect on species’ abundances; (d) the effect of assuming time-varying effects of temperature niche and habitat breadth (effect sizes assumed to increase and decrease over time respectively) in the underlying dynamics of the simulated species and (e) the effect of including random variation in the density-dependence of the underlying dynamics of the simulated species.
