# Supplementary material for: Improving the community-temperature index as a climate change indicator
Source: PLoS One. 2017 Sep 12;12(9):e0184275. doi: 10.1371/journal.pone.0184275 (PMC5595310; doi:10.1371/journal.pone.0184275)
Supplement: S1 Table — (DOCX) [file pone.0184275.s006.docx]

**S1 Table** Definition of community temperature index (CTI) terminology

| Term | A weighted average of species’ temperature preferences, with weighting by: |
| --- | --- |
| Original CTI | Observed counts of each species |
| True CTI | Observed counts of each species - in a fully known system in which only temperature preference affects population growth – it is only applicable to a simulation scenario |
| Modelled CTI | Predicted counts of each species – in which year-to-year changes in counts are only predicted using coefficients related to temperature preference |
| Modelled CTI - uncorrected | Predicted counts of each species – in which year-to-year changes in counts are predicted using coefficients related to temperature preference and other species attributes |
