# Supplementary material for: Improving the community-temperature index as a climate change indicator
Source: PLoS One. 2017 Sep 12;12(9):e0184275. doi: 10.1371/journal.pone.0184275 (PMC5595310; doi:10.1371/journal.pone.0184275)
Supplement: S1 File — (DOCX) [file pone.0184275.s007.docx]

#########################################################################

#Code to supplement: Bowler et al. A model-based approach to calculating #the community-temperature index: accounting for covariation among #species attributes

#########################################################################

#Section 1: Code to simulate the effects of covariation between species #temperature niche and other species attributes on the estimation of the #Community Temperature Index (CTI), and to compare the standard CTI #calculation with our proposed model-based CTI calculation.

#########################################################################

#run simulated dynamics 500 times

for(run in 1:500){

#########################################################################

#libraries needed

library(INLA)

library(plyr)

library(Hmisc)

library(reshape2)

library(MASS)

#########################################################################

#assume we have a community of 50 species

Species<-gl(50,1)

j.species<-length(Species)

#generate two Normally distributed correlated variables that will become #our species attributes code based on: #http://stats.stackexchange.com/questions/15011/generate-#a-random-#variable-with-a-defined-correlation-to-an-existing-variable

n <- 50

rho <- 0.6 # desired correlation

theta <- acos(rho)

x1 <- rnorm(n, 1, 1)

x2 <- rnorm(n, 1, 1)

X <- cbind(x1, x2)

Xctr <- scale(X, center=TRUE, scale=FALSE)

Id <- diag(n)

Q <- qr.Q(qr(Xctr[ , 1, drop=FALSE]))

P <- tcrossprod(Q) # = Q Q'

x2o <- (Id-P) %*% Xctr[ , 2]

Xc2 <- cbind(Xctr[ , 1], x2o)

Y <- Xc2 %*% diag(1/sqrt(colSums(Xc2^2)))

x <- Y[ , 2] + (1 / tan(theta)) * Y[ , 1]

#assign these variables as habitat specialism (HS, i.e, inverse of #habitat breadth)and temperature niche (TN)

HS<-scale(x1)

TN<-scale(x)

#additionally, resample the temperature niche variable to create an #uncorrected variable (TNunc)

TNunc<-sample(TN)

#Make a data frame of these species attributes

species.df<-data.frame(Species,HS,TN,TNunc)

#specify the effect of species attributes on species growth rates

#habitat specialism has a negative effect (assumed caused by land use #change), and temperature niche has a positive effect (assumed caused by #climate change)of equal magnitude

HS.effect<--0.05

TN.effect<-0.05

#create matrix to hold species abundances

x<-matrix(ncol=50,nrow=20)

#initialise each population with mean size of 50 individuals

x[1,]<-rpois(50,50)

#########################################################################

#Simulations (1) "true CTI" - without any effect of habitat breadth and #no covariation

#project the growth of each species onwards for 20 years

for(j in 1:j.species){

for(i in 2:20){

x[i,j]<-log(x[i-1,j])+TN.effect*TN[j]

x[i,j]<-rpois(1,exp(x[i,j]))

}}

#melt data frame

x1<-data.frame(x)

names(x1)<-Species

x1$Year<-1:20

df<-melt(x1,id=c("Year"))

names(df)[2:3]<-c("Species","Count")

df<-merge(df,species.df,by="Species")

#calculate the true CTI

CTIanalysis1<-ddply(df,.(Year),summarise,CTI=wtd.mean(TN,weights=Count))

#########################################################################

#Simulations (2) No covariation between temp niche and habitat breadth

#but habitat breadth affects species growth

for(j in 1:j.species){

for(i in 2:20){

x[i,j]<-log(x[i-1,j])+HS.effect*HS[j]+TN.effect*TNunc[j]

x[i,j]<-rpois(1,exp(x[i,j]))

}}

#melt data frame

x2<-data.frame(x)

names(x2)<-Species

x2$Year<-1:20

df<-melt(x2,id=c("Year"))

names(df)[2:3]<-c("Species","Count")

#get uncorrected CTI

CTIanalysis2_uncorr<-ddply(df,.(Year),summarise,CTI=wtd.mean(TNunc,weights=Count))

#now get corrected CTI

#create variables for the population model

df$Species.int<-as.numeric(df$Species)

df$Species.idx<-df$Species.int+max(df$Species.int)

df$fYear<-as.factor(df$Year)

df<-merge(df,species.df,by="Species")

#fit model

inla1<-inla(Count~fYear*HS+fYear*TNunc+f(Species.int,model="iid")+f(Year,model="ar1"),data=df,family="poisson",control.predictor=list(compute=T,link=1))

#get model fits just using temperature niche as the coefficient affecting #year-to-year change

outFE<-data.frame(as.matrix(inla1$model.matrix))

myvars<-names(outFE)[!(grepl("fYear",names(outFE))&!grepl("TN",names(outFE)))]

outFE[,!names(outFE)%in%myvars]<-0#those relating to change over time and #not temp niche set to zero

outFE<-as.matrix(outFE)

coefFE<-as.matrix(inla1$summary.fixed[,1],ncol=1)

df$mypreds<-outFE%*%coefFE

##also get species random intercepts term and add it on

temp<-inla1$summary.random[["Species.int"]][,c("ID","mean")]

df$speciesint<-temp$mean[match(df$Species.int,temp$ID)]

df$mypreds<-df$mypreds+df$speciesint

#get corrected CTI based on these corrected fits

CTIanalysis2_corr<-ddply(df,.(Year),summarise,CTI=wtd.mean(TNunc,weights=exp(mypreds)))

#########################################################################

#Simulations (3) Covariation between temp niche and habitat breadth

#and habitat breadth affects species growth

for(j in 1:j.species){

for(i in 2:20){

x[i,j]<-log(x[i-1,j])+HS.effect*HS[j]+TN.effect*TN[j]

x[i,j]<-rpois(1,exp(x[i,j]))

}}

#melt data frame

x3<-data.frame(x)

names(x3)<-Species

x3$Year<-1:20

library(reshape2)

df<-melt(x3,id=c("Year"))

names(df)[2:3]<-c("Species","Count")

#get uncorrected CTI

CTIanalysis3_uncorr<-ddply(df,.(Year),summarise,CTI=wtd.mean(TN,weights=Count))

#now get corrected one

#create variables for the population model

df$Species.int<-as.numeric(df$Species)

df$Species.idx<-df$Species.int+max(df$Species.int)

df$fYear<-as.factor(df$Year)

df<-merge(df,species.df,by="Species")

#fit model

inla1<-inla(Count~fYear*HS+fYear*TN+f(Species.int,model="iid")+f(Year,model="ar1"),data=df,family="poisson",control.predictor=list(compute=T,link=1))

#get model fits just using temperature niche as the coefficient affecting #year-to-year change

outFE<-data.frame(as.matrix(inla1$model.matrix))

myvars<-names(outFE)[!(grepl("fYear",names(outFE))&!grepl("TN",names(outFE)))]

outFE[,!names(outFE)%in%myvars]<-0#those relating to change over time and not temp niche set to zero

outFE<-as.matrix(outFE)

coefFE<-as.matrix(inla1$summary.fixed[,1],ncol=1)

df$mypreds<-outFE%*%coefFE

#also get species random intercepts and add it on

temp<-inla1$summary.random[["Species.int"]][,c("ID","mean")]

df$speciesint<-temp$mean[match(df$Species.int,temp$ID)]

df$mypreds<-df$mypreds+df$speciesint

#get corrected CTI based on these corrected fits

CTIanalysis3_corr<-ddply(df,.(Year),summarise,CTI=wtd.mean(TN,weights=exp(mypreds)))

#########################################################################

#bring together

CTIanalysis1$Type<-"true CTI"

CTIanalysis2_uncorr$Type<-"Uncorrected CTI (no covariation)"

CTIanalysis2_corr$Type<-"Corrected CTI (no covariation)"

CTIanalysis3_uncorr$Type<-"Uncorrected CTI (covariation)"

CTIanalysis3_corr$Type<-"Corrected CTI (covariation)"

CTIanalysis<-rbind(CTIanalysis1,CTIanalysis2_uncorr,CTIanalysis2_corr, CTIanalysis3_uncorr,CTIanalysis3_corr)

#qplot(Year,CTI,data=CTIanalysis,geom="line")+facet_wrap(~Type,nrow=1)

save(CTIanalysis,file=paste0("CTIrun_",run,".RData"))

}

#########################################################################

#########################################################################

#Section 2: Running analysis on a real data set- calculating both the #original CTI and the model-based approach

#########################################################################

#The community data is assumed to be in a data frame called "df" that #contains the following columns:

#"Species" - species name

#"Year" - the year that the count data pertains to

#"Count" - the number of individuals of the species seen in that year

#"TMean" - the temperature niche of the species

#"Habitat" - the habitat preference of the species

#standard way of calc CTIs

CTIorig<-ddply(df,.(Year),summarise,annualCTI=wtd.mean(TMean,weights=Count))

qplot(Year,annualCTI,data=CTI,geom=c("line","point"))+ylab("CTI")+xlab("Year")+theme_bw()

CTIorig$Type<-"Original CTI"

#model-based approach to calculating the CTI

#if necessary insert NAs in the count column for years when there is #missing count data as follows:

newgrid<-unique(data.frame(df[,c("Species","TMean","Habitat")]))

dfI<-ldply(unique(df$Year),function(x)cbind(x,newgrid))

names(dfI)[1]<-"Year"

df<-merge(dfI,df,all.x=T)

#create index variables for the INLA population model

df$Year<-as.numeric(df$Year)

df$iYear<-df$Year-min(df$Year)+1

df$fYear<-as.factor(df$Year)

df$Species<-factor(df$Species)

df$Species.int<-as.numeric(as.factor(df$Species))

df$Species.idx<-df$Species.int+max(df$Species.int)

#fitting population model with INLA - see http://www.r-inla.org/

library(INLA)

inla1<-inla(log(Count)~fYear*TMean+fYear*Habitat+

#f(iYear,model="ar1",replicate=Species.idx,constr=T)+ #allowing variation in ar1 among species?

f(iYear,model="ar1")+

f(Species.int,model="iid"),

data=df,

control.predictor=list(compute=T,link=1))

#get fits - just using temperature niche for those affecting change over #time

outFE<-data.frame(as.matrix(inla1$model.matrix))

myvars<-names(outFE)[!(grepl("fYear",names(outFE))&!grepl("TMean",names(outFE)))]

outFE[,!names(outFE)%in%myvars]<-0#those relating to change over time and #not temp niche set to zero

outFE<-as.matrix(outFE)

coefFE<-as.matrix(inla1$summary.fixed[,1],ncol=1)

df$mypreds<-outFE%*%coefFE

##also get species random intercepts and add it on

temp<-inla1$summary.random[["Species.int"]][,c("ID","mean")]

df$speciesint<-temp$mean[match(df$Species.int,temp$ID)]

df$mypreds<-df$mypreds+df$speciesint

#calc CTIs

CTIcorr<-ddply(df,.(Year),summarise,annualCTI=wtd.mean(TMean,weights=exp(mypreds)))

CTIcorr$Type<-"Corrected CTI"

#Combine and plot the CTIs

allCTI<-rbind(CTIorig,CTIcorr)

qplot(Year,annualCTI,data=allCTI,colour=Type,geom="blank")+

geom_line(size=1.2,aes(linetype=Type))+theme_bw()+

xlab("Year")+ylab("Community Temperature Index")+

scale_colour_manual(values = c("limegreen","steelblue4"))+

theme(legend.position="top")

#########################################################################
